# Olesoxime (TRO19622): A Novel Mitochondrial-Targeted Neuroprotective Compound

**DOI:** 10.3390/ph3020345

**Published:** 2010-01-28

**Authors:** Thierry Bordet, Patrick Berna, Jean-Louis Abitbol, Rebecca M. Pruss

**Affiliations:** Trophos, Parc Scientifique de Luminy, Case 931, 13288 Marseille cedex 9, France; E-Mails: tbordet@trophos.com (T.B.); pberna@trophos.com (P.B.); jlabitbol@trophos.com (J.L.A.)

**Keywords:** TRO19622, olesoxime, neuroprotection, mitochondrial permeability transition pore, motor neuron disease, amyotrophic lateral sclerosis, spinal muscular atrophies

## Abstract

Olesoxime (TRO19622) is a novel mitochondrial-targeted neuroprotective compound undergoing a pivotal clinical efficacy study in Amyotrophic Lateral Sclerosis (ALS) and also in development for Spinal Muscular Atrophy (SMA). It belongs to a new family of cholesterol-oximes identified for its survival-promoting activity on purified motor neurons deprived of neurotrophic factors. Olesoxime targets proteins of the outer mitochondrial membrane, concentrates at the mitochondria and prevents permeability transition pore opening mediated by, among other things, oxidative stress. Olesoxime has been shown to exert a potent neuroprotective effect in various *in vitro* and *in vivo* models. In particular olesoxime provided significant protection in experimental animal models of motor neuron disorders and more particularly ALS. Olesoxime is orally active, crosses the blood brain barrier, and is well tolerated. Collectively, its pharmacological properties designate olesoxime as a promising drug candidate for motor neuron diseases.

## 1. Introduction

Despite the identification of specific genes and proteins that are invariably associated with neurodegenerative disorders, there are currently no validated molecular targets and no effective therapies to slow or cure these devastating diseases. To avoid the pitfalls of target-based drug discovery, phenotypic screening using primary neurons offers the opportunity to select compounds active in a relevant pathophysiological context and eventually identify and validate new drugable targets. This hypothesis was the basis of the strategy to develop cell based assays using primary motor neurons to identify drug candidates for amyotrophic lateral sclerosis (ALS) and spinal muscular atrophy (SMA) [1]. Using primary neurons that express relevant target(s) under the conditions that lead to their dysfunction or death, such as excitotoxicity or trophic factor withdrawal it is possible to define a simple assay endpoint based on cell survival. The relevance and power of such a simple bioassay is demonstrated by the fact that it is the basis for the identification of all known neurotrophic factors. Therefore, identification of small molecules providing similar survival benefits should be possible.

However, several factors make such a simple assay system difficult to realize. First, culturing primary neurons requires specialized know how. Second, the yield is generally low, limiting screening throughput. Third, this "black box" approach has been considered challenging for chemists to develop coherent structure activity relationships in order to optimize drug candidates. Despite these issues, we have used phenotypic screening to identify a novel family of “cholesterol oximes” that promotes the survival of trophic factor deprived primary rat motor neurons. 

Using standard binding, electrophysiology and enzyme assays we found that these compounds are devoid of activity on classical drug targets but bind to mitochondrial outer membrane proteins implicated in oxidative stress-induced mitochondrial permeability transition and apoptotic factor release. This suggested that their cytoprotective properties may be due at least in part to improvement of mitochondrial dysfunction. Since mitochondrial dysfunction is now recognized as a factor in nearly all neurodegenerative diseases as well as in other non-neurological conditions such as cardiac reperfusion injury, we validated this hypothesis by demonstrating activity in relevant preclinical models of these disorders. 

Here we describe the paths leading to the discovery and development of olesoxime, a compound now in clinical trials for the treatment of ALS and SMA. This example shows that in spite of the potential difficulties associated with phenotypic screening, there may be some advantages: compounds identified are relatively stable (since assays take days, not h, to perform), non-toxic (since cell survival is the endpoint) and can cross the cell membranes (to interact with mitochondrial targets) so even “hits” in such assays fulfill many of the criteria necessary to declare a lead molecule a drug candidate. Furthermore, standard chemical strategies to define SAR and improve properties as may be required for future clinical development are indeed possible and have lead to other novel family members now in development for cardiovascular indications. 

## 2. Discovery of Olesoxime

### 2.1. Chemistry

**Figure 1:**
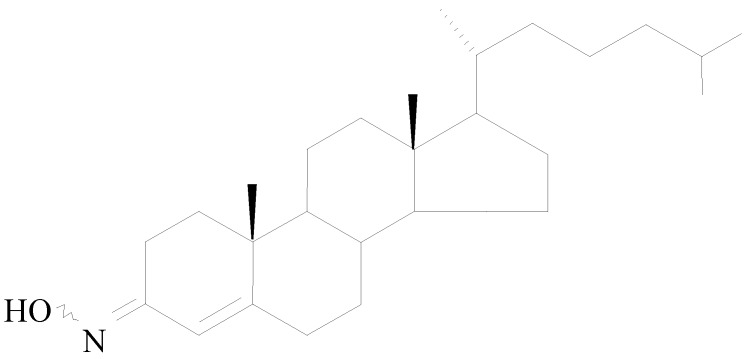
Olesoxime chemical structure.

Olesoxime (Trophos code: TRO19622; chemical name: cholest-4-en-3-one, oxime) is a cholesterol-like compound with a molecular weight of 399.65 Da (C_27_H_45_NO) that exists as a stable mixture of *syn* and *anti* isomers of the oxime in the 3-position (Figure 1). Olesoxime as a crystalline powder is stable for more than 36 months under conditions described in regulatory guidelines. Due to its lipophilic properties, oily excipients can be used to prepare solid and liquid oral dosage formulations for preclinical and clinical studies.

### 2.2. Molecular Pharmacology

Olesoxime was initially identified based on its survival-promoting activity on purified cultured rat motor neurons deprived of neurotrophic factors [2]. Because of its cholesterol-like structure, solubility in aqueous media is limited but can be facilitated by addition of a small amount of serum or albumin. In a nearly serum-free medium, olesoxime has similar efficacy as a cocktail of three neurotrophic factors (brain derived neurotrophic factor, cilliary neurotrophic factor and glial-cell-line-derived neurotrophic factor) used as a positive control for maximal survival and neurite outgrowth in the motor neuron screen with potency in the low micromolar range (EC50 = 3 µM; further detailed below). 

Wide ranging pharmacological profiling did not reveal interactions with a large panel of physiologically important targets: nuclear steroid hormone receptors, enzymes, ion channels, neurotransmitter receptors or transporters. However, investigation of unconventional steroid- or cholesterol-binding targets revealed that olesoxime binds to two outer mitochondrial membrane proteins, TSPO 18kDa (formerly referred to as the Peripheral Benzodiazepine Receptor or PBR) and the voltage-dependent anion channel (VDAC). As these two proteins have been implicated in the regulation of mitochondrial metabolism, response to oxidative stress and modulation of mitochondrial permeability transition, it seemed logical to consider mitochondria as the relevant target for olesoxime’s pro-survival activities. This implicated that olesoxime could be beneficial to other classes of neurons besides motor neurons or even non-neuronal cells. To indirectly confirm or refute this conjecture, olesoxime was studied in a wide range of cellular and animal models of neurodegeneration.

## 3. Neuroprotection

### 3.1. In Vitro Neuroprotection

**Figure 2:**
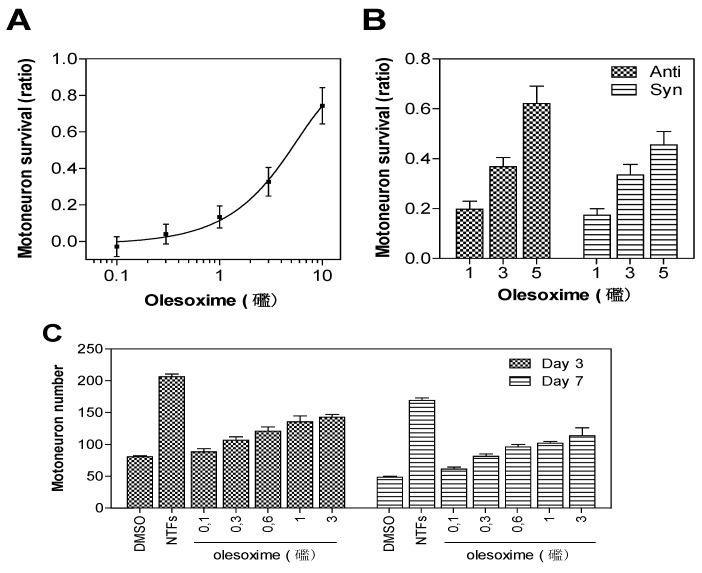
Neuroprotective dose-effect of olesoxime against motor neuron cell death. Rat embryonic motor neurons were cultured in 96-well plates in the absence of trophic factors and with increasing concentrations of olesoxime (A, C), or with pure syn or anti forms of olesoxime (B). Motor neuron survival was measured after 3 days (A-C) or 7 days (C) *in vitro* by direct counting of live cells labelled with calcein-AM. Survival is expressed as a ratio of surviving cell (A-B) relative to positive controls treated with a cocktail of neurotrophic factors (NTFs) and negative controls that received DMSO alone (0.1%). Data represent mean ± SEM.

Rat embryonic motor neurons undergoing trophic factor deprivation are a well-defined *in vitro* model system for studying neuronal cell death mechanisms [3]. Trophic factor-deprivation leads to rapid generation of reactive oxygen species (ROS), neuronal nitric oxide synthase induction, mitochondrial dysfunction and loss of energy production that culminate in neuronal cell death [4,5]. Besides their effects on neuronal survival, trophic factor signalling is important for neuronal polarization and neurite outgrowth (for review see [6]). Since early breakdown of neuromuscular junctions could reduce the supply of muscle-derived neurotrophic factors required for neurite outgrowth and survival of spinal motor neurons, an *in vitro* model based on primary motor neuron survival was considered to be a relevant phenotypic screening assay for motor neuron diseases; this assay has identified all known neurotrophic factors. E14 rat motor neurons were cultured in the absence of trophic factors with or without olesoxime. After three days of culture, cell survival was determined by counting live motor neurons able to take up and convert calcein-AM to its fluorescent product. Trophic factor deprivation results in about 50% decrease in cell survival when compared to controls supplemented with trophic factors. Treatment with olesoxime at concentrations ranging from 0.1 to 10 µM in 0.1% DMSO produced a dose-dependent increase in cell survival with EC_50_ around 3 µM [2]. Importantly, both syn and anti forms were equally potent. Survival benefit was maintained for up to seven days after a single addition of olesoxime, indicating a long-lasting neuroprotective effect for primary motor neurons (Figure 2).

**Figure 3:**
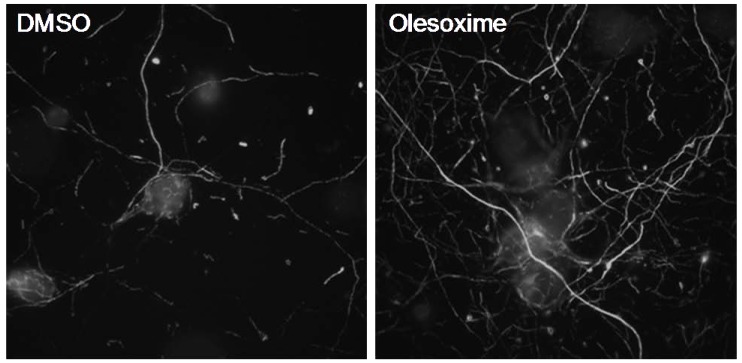
Olesoxime promotes neurite outgrowth of rat primary cortical neurons. E18 cortical neurons were cultured for 6 days in Neurobasal medium, 2% B27 (Invitrogen) and 1% pyruvate. After 24 h treatment with 5µM olesoxime, an increased density in neurite network was observed by immunostaining of neurofilaments.

To extend these observations and explore the general nature of its pro-survival effects, we studied the effects of olesoxime in several other neuronal cell-based assays (Table 1). The broad neuroprotective effects on various classes of neurons and neurodegeneration models, were consistent with the hypothesis that olesoxime targets a common mechanism of cell death. Along with its neuroprotective potential, olesoxime also displays regenerative activities by promoting neurite outgrowth by cultured rat motor neurons (supplementary data in [2]) and rat cortical neurons (Figure 3).

**Table 1:** Neuronal cell-based assays in which olesoxime displayed neuroprotective activities.

Cell type	Stress condition	Outcome
Motor neuron	Trophic factor deprivation [2]	Neuronal survival and neurite outgrowth
Striatal neuron	Mutant Htt overexpression [7]	Neuronal survival
Cerebellar granule neuron	Low K+ [2]	Decreased cyt c release and neuronal survival
Cortical neuron	Camptothecin-induced toxicity (unpublished data)	Decreased cyt c release and neuronal survival

### 3.2. In Vivo Neuroprotection—Nerve Lesion Models

#### 3.2.1. Neonatal Motor Neuron Axotomy

Axotomy of the facial nerve in neonatal rats is a well-defined *in vivo* model system to induced programmed cell death of motor neurons [8,9,10]. When performed on 2 day-old rats, facial nerve axotomy resulted in the death of 80% of the motor neurons in the facial motor nucleus [11] that can be partially rescued by application of mitochondrial pore inhibitors [12]. Treatment with olesoxime by oral gavage 4 h before nerve axotomy and then once a day for 5 days, at 10 and 100 mg/kg formulated in hydroxypropyl methylcellulose, increased the number of surviving motor neurons by 20% and 40%, respectively (P < 0.01 at the highest dose). Similar protection as found with the higher dose was observed with oral administration of olesoxime at 30 mg/kg when it was dissolved in vegetable oil [2].

#### 3.2.2. Peripheral Nerve Trauma

In a second model of nerve lesion, the effect of olesoxime was investigated on the axonal degeneration induced by the crush of the sciatic nerve in mice. In this animal model, the extent and speed of nerve regeneration is usually defined by the return of nerve function [13]. The activity of olesoxime was evaluated in this model after subcutaneous administration of 0.3, 3, and 30 mg/kg/day in CDEP (cremophore EL/DMSO/ethanol/phosphate buffer saline; 5/5/10/80% respectively). Sciatic nerve crush was performed on 8 week-old female C57BL/6 RJ mice and compound treatment started the same day and was repeated each day during 6 weeks. The nerve degeneration/regeneration process was followed by measurements of the compound muscular action potential (CMAP) and histological studies of the damage area of the sciatic nerve. Treatment with olesoxime (3 or 30 mg/kg sc) reduced the proportion of degenerated fibers in lesioned nerves by 69% as compared to animals receiving vehicle treatment. Moreover, olesoxime accelerated nerve regeneration: improvements were observed in neuro-muscular function starting at week 2 post-lesion, with maximum recovery of CMAP amplitude and CMAP latency from week 4 to week 6. This is in accordance with the histological analysis of nerve samples showing a clear overall increase in the axonal size (mean axonal size ± SEM: 7.6 ± 0.1 µm² in the olesoxime 30 mg/kg group *versus* 6.0 ± 0.1 µm² in the vehicle group, p < 0.05) along with an enhanced myelination. Thus, olesoxime reduces fiber degeneration and improves nerve fiber regeneration correlating with previous observations in *in vitro* neuronal cultures.

### 3.3. In Vivo Neuroprotection—Chronic Motor Neuron Disease Mouse Models

Both amyotrophic lateral sclerosis (ALS) and spinal muscular atrophy (SMA) are progressive and fatal motor neuron diseases leading to progressive denervation and atrophy of skeletal muscles, paralysis and most often death. Although the upstream factors that trigger ALS and SMA are diverse, there is now clear evidence for the role of neuromuscular junction denervation, axonal degeneration and programmed cell death in both diseases (see recent reviews [14,15,16]). The clinical relevance of blocking these processes has been demonstrated by studies in animal models. These have shown that administration of exogenous survival (neurotrophic) factors [17,18], or inhibition of endogenous cell death pathways [19,20,21], can lead to increased survival in animal models used to predict potential clinical benefit.

#### 3.3.1. SOD1G93A Transgenic Mouse Model of ALS

In about 90% of the cases the cause of ALS is unknown. However, of the 10% of familial cases, nearly 20% are associated the cytoplasmic Cu2+/Zn2+ superoxide dismutase (SOD1) gene, for which more than 100 mutations are known. Transgenic mice overexpressing the G93A mutated form of the human SOD1 gene are a commonly used model for familial ALS [22]. As already reported by others, from week 15 onwards SOD1G93A mice displayed a progressive decrease in body weight, along with impaired performance on the rotorod or grid test indicative of a decline in motor coordination and median age at death was around 135 days of age. Litters from heterozygous, confirmed high SOD1G93A copy number, G1H males (supplied by Jackson labs) bred to B6SJL F1 females were randomized into 4 groups with approximately even numbers of each gender per group. Mice were treated daily by subcutaneous injection of 3 or 30 mg/kg olesoxime in CDEP or its vehicle starting at 60 days of age until the end of their life. Only deaths due to “ALS” e.g., paralysis, were counted for the survival endpoint. Olesoxime treatment with the dose of 3 mg/kg (the minimal fully effective dose in the sciatic nerve crush model) prevented the weight loss that occurs in the SOD1G93A mice before death (two-way ANOVA test from D106 to D135, p < m0.01) and both doses led to a significant delay in the decline in performance on the grid test and rotorod (two-way ANOVA test from week 14 to week 21, p < 0.05) suggesting a delay in disease onset. Finally, olesoxime significantly increases survival of SOD1G93A mice (138 ± 4 days in olesoxime 3 mg/kg/day group versus 125 ± 3 days in vehicle-treated animals; t-test p = 0.009, log rank p = 0.018). Since the delay in onset was similar in magnitude to the prolongation of lifespan, we concluded that the major beneficial effect of olesoxime was on disease onset rather than progression [2]. Riluzole, which has been validated to provide a clinically meaningful increase in survival, was used as a positive control but the dose tested (8 mg/kg, i.p.) had no effect on motor performance and resulted in only a small but insignificant increase in mean survival time (t-test p = 0.74; log rank test p = 0.50).

#### 3.3.2. A Transgenic Mouse Model of SMA

Spinal muscular atrophy (SMA) is the second most frequent autosomal recessive genetic disease in humans with an incidence of approximately one in 6,000 births [23,24,25]. It is caused by the homozygous loss of the telomeric copy of the survival motor neuron gene (*SMN1*) on human chromosome 5q13 [26,27]. Insufficient levels of the SMN protein coming from a nearly identical SMN2 gene result in neuromuscular junction degeneration, selective loss of spinal motor neurons, progressive muscle weakness and ultimately respiratory failure [28,29]. In the mouse genome, there is one *Smn* gene [30,31] and its deletion results in embryonic lethality [32,33,34]. Its specific deletion in neurons on mutant NSE-Cre; SMNF7/F7 mice led to progressive motor defects and neuromuscular junction alterations suggestive of a ‘dying-back’ degenerative process in SMA [34,35]. In this severe model, daily treatment with olesoxime by subcutaneous injection at 30 mg/kg, starting at 21 days of age until the end of their life, induced a significant increase in life span (log rank test, p < 0.05). Only 15% of vehicle-treated animals survived for longer than 40 days whereas 45% of the olesoxime-treated mice were still alive after 40 days. 

### 3.4. In Vivo Neuroprotection—Painful Peripheral Neuropathies Models

Olesoxime significantly reduced axonal degeneration and accelerated recovery of motor nerve conduction in a model of peripheral neuropathy induced by crushing the sciatic nerve motivating further exploration in other preclinical models of peripheral neuropathy.

#### 3.4.1. Chemotherapy-Induced Peripheral Neuropathy (CIPN)

CIPN is a major dose and treatment limiting side effect for cancer patients who receive multiple cycles of platins and microtubule targeting drugs. CIPN can persist long after the cancer treatment has stopped and has a serious impact on quality of life. Mitochondrial dysfunction may be an important factor underlying the development of CIPN, particularly in the case of microtubule targeted drugs. Although high doses of paclitaxel or vincristine disrupt neuronal microtubules leading to axonal degeneration [36,37], neuropathic pain behavior appears in rats even in the absence of degeneration at the level of the peripheral nerve [36,37]. Similarly, peripheral nerve biopsies from neuropathic pain patients induced by vincristine treatment revealed axonal and mitochondrial swelling while there was no evidence of microtubule alterations [38].

In rats treated for one week with paclitaxel (2 mg/kg, i.p., on day 0, 2, 4, and 6), the slowly developing but persistent pain is correlated with the presence of swollen axons and vacuolated mitochondria in peripheral nerve, abnormal spontaneous discharge and partial degeneration of intraepidermal sensory nerve arbors (IENF) [39,40,41,42,43,44]. Prophylactic dosing from day -1 to day 15 with either 3 mg/kg or 30 mg/kg olesoxime significantly prevented the loss of IENF in the paw of paclitaxel-treated rats (317.3 ± 7.6 IENFs per cm in naïve animal, 172.0 ± 14.3 in paclitaxel-treated animals receiving vehicle, 238.5 ± 17.6 and 247.2 ± 14.4 in 3 mg/kg and 30 mg/kg olesoxime groups, respectively). Both doses of olesoxime also significantly (p < 0.01) inhibited the development of mechano-allodynia and mechano-hyperalgesia following paclitaxel treatment [45]. These results suggest that olesoxime treatment may be clinically useful as a prophylactic treatment for both paclitaxel-evoked neuropathy and the neuropathic pain syndrome that sometimes accompanies it. The feasibility of such use is supported by preliminary results showing that olesoxime does not interfere with the anti-mitotic or cytotoxic effects of paclitaxel on proliferating tumor cell lines (unpublished data).

#### 3.4.2. Diabetes-Induced Peripheral Neuropathy (DNP)

Streptozotocin (STZ) administration induces diabetes in rats that evolves to include a painful neuropathy mimicking the clinical condition [46,47,48,49,50,51]. Neuropathy in this animal model is associated with decreased nerve blood flow, axonal atrophy of both the motor and sensory nerve fibers, slowing of nerve conduction velocity [52,53] and hyperexcitability of C-fibers [54,55,56]. Daily treatment with olesoxime for 30 days, starting 10 days post-STZ, significantly improved motor nerve conduction in STZ-diabetic rats as measured by a reduction in compound muscle action potential latency. Furthermore, besides its neuroprotective effects, olesoxime also reversed tactile allodynia after repeated administration in STZ-induced diabetic rats [57].

To conclude, olesoxime showed significant neuroprotective and neuroregenerative effects in animal models of motor nerve degeneration as well as anti-nociceptive and neuroprotective effects in experimental models of painful peripheral neuropathies induced by chemotherapy or diabetes. Table 2 summarizes these pharmacological activities and the corresponding active concentrations of olesoxime in these different animal models. These results demonstrate that chronic exposure to circulating plasma concentrations as low as 0.15 µM are able to delay neurodegeneration and promote neuroregeneration although higher concentrations (>1.5 µM) are needed to reverse neuropathy-induced pain.

**Table 2:** Summary of *in vivo* pharmacological activities of olesoxime.

Model		Route	Treatment (days)	MED (mg/kg)	Css,_max _(µg/mL)
Axotomy model		PO	5	30	20
Nerve crush model		SC	42	3	0.6
SMA transgenic mice		SC	D21 to death	30	12
ALS transgenic mice		SC	D60 to death	3	0.6
Paclitaxel-induced NP in rat	Prevention of IEFNs degeneration	PO	17	< 3	0.16
	Treatment of mechano-allodynia & hyperalgesia	PO	5	10	0.5
STZ diabetic rat	Improvement in motor nerve conduction	PO	32	3	0.3
	Treatment of tactile allodynia	PO	5	10	1.8

Subcutaneous (SC) injection were performed in Cremophore EL/dimethylsulfoxide/ethanol /Phosphate Buffer Saline (5/5/10/80 v/v); per os (PO) administration were performed by oral gavage in vegetable oil. MED, minimal effective dose; Css max, mean maximal concentration at steady state values determined from parallel satellite animals; IEFNs, intraepidermal nerve fibers.

## 4. Further Evidence that Olesoxime Targets Mitochondria

### 4.1. Olesoxime Concentrates at the Mitochondria

**Figure 4:**
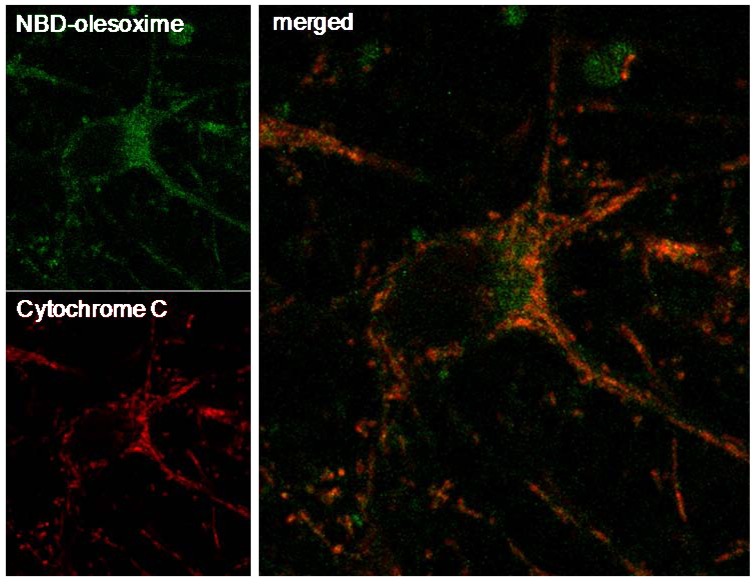
Mitochondrial localization of NBD-olesoxime in primary cortical neurons. Rat E18 cortical neurons were treated with 10µM NBD-olesoxime (green) for 24 h, then immunostained for cytochrome C (red) and processed for confocal imaging. The orange staining in the merged image demonstrated localization of olesoxime in mitochondria.

Previous observations revealed that olesoxime binds to two outer mitochondrial membrane proteins, TSPO 18kDa and VDAC making it logical to consider mitochondria as the relevant target for olesoxime. Further evidence of the mitochondrial targeting of olesoxime was provided by the co-localization of cytochrome C and a fluorescent analogue of olesoxime (NBD-Olesoxime or 3-oxyimino-25-[méthyl(7-nitro-2,1,3-benzoxadiazol-4-yl)amino]-27-norcholest-4-ène) in mitochondria of primary cortical neurons (Figure 4).

### 4.2. Olesoxime Does Not Increase Calcium Retention Capacity in Isolated Mitochondria

As olesoxime binds to proteins implicated in the formation or regulation of the mitochondrial permeability transition pore (mPTP), it was tempting to speculate that its neuroprotective properties could arise from the modulation of the mPTP. The sustained opening of the mPTP results in an increase in mitochondrial membrane permeability that leads to the loss of mitochondrial membrane potential, alteration in calcium fluxes, mitochondrial swelling, rupture of the outer mitochondrial membrane and the release of pro-apoptotic factors including cytochrome c and apoptosis-inducing factor (reviewed in [58,59]). Cyclosporine A (CsA) potently inhibits mPTP by targeting cyclophilin D in the mitochondrial matrix. *In vitro*, CsA increases mitochondrial calcium retention capacity and calcium-induced swelling of isolated mitochondria [60].

**Figure 5:**
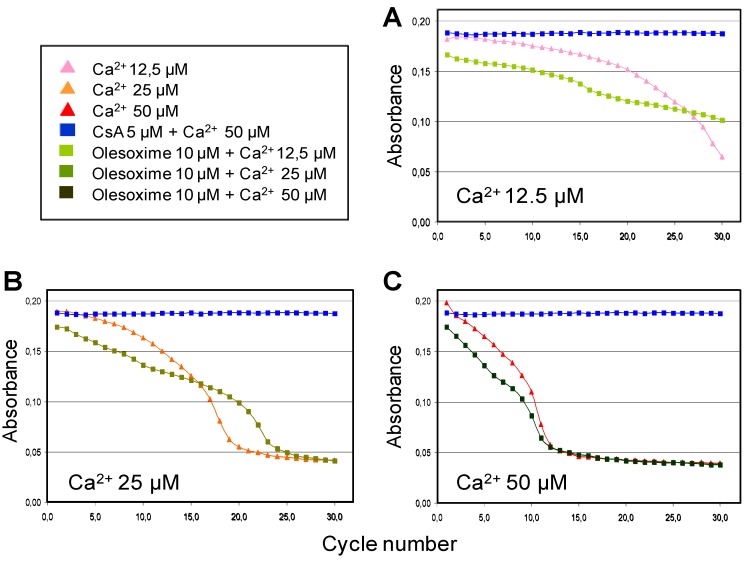
Partial effects of olesoxime on calcium-induced mitochondrial swelling. Mitochondria purified from mouse liver were incubated with 10 µM olesoxime or 5 µM CsA for 5 minutes, prior to the induction of swelling by different calcium concentrations (**A**, 12.5 µM; **B**, 25 µM; **C**, 50 µM). Thereafter, mitochondrial swelling was monitored every 2 min by determining the light scattering or decreased absorbance of the samples.

The ability of olesoxime to inhibit calcium-induced mPTP opening and swelling of isolated liver mitochondria showed that olesoxime had only a small effect compared to CsA and only slightly delayed swelling when low doses of calcium were used to induce mPTP opening (Figure 5). Similarly, olesoxime did not induce any changes in calcium retention capacity of freshly prepared mouse hepatic mitochondria (Table 3). Thus olesoxime did not modulate calcium-induced mitochondrial permeability transition assayed using isolated liver mitochondria.

**Table 3:** Calcium retention capacity of isolated mitochondria. Freshly prepared mitochondria from mouse liver were incubated with increasing concentrations of olesoxime or CsA for 5 minutes prior to the start of calcium addition. Pulses of 10 µM calcium were added and Calcium Green fluorescence was measured 50 seconds after addition to measure extramitochondrial calcium concentration until there was no further mitochondrial uptake. The number of calcium pulses prior to loss of calcium uptake was used to calculate the mitochondrial calcium retention capacity. Data represent mean ± SD (2 independent experiments run in triplicate each).

μM	Control	Olesoxime				CsA			
		0.1	0.5	1	5	0.1	0.5	1	5
mPTP induction (μM Ca^2+^)	34.5	36.6	37.9	35.4	35.7	71.4	80.7	85.3	87.7
SD	1.72	0.80	2.28	1.04	0.57	1.12	3.48	1.25	2.71
Norm. *vs* control	1	2.1	3.4	0.8	1.2	36.9	46.2	50.8	53.1

### 4.3. Olesoxime Rescues Cardiomyocytes from Doxorubicin-Induced PTP Opening

**Figure 6:**
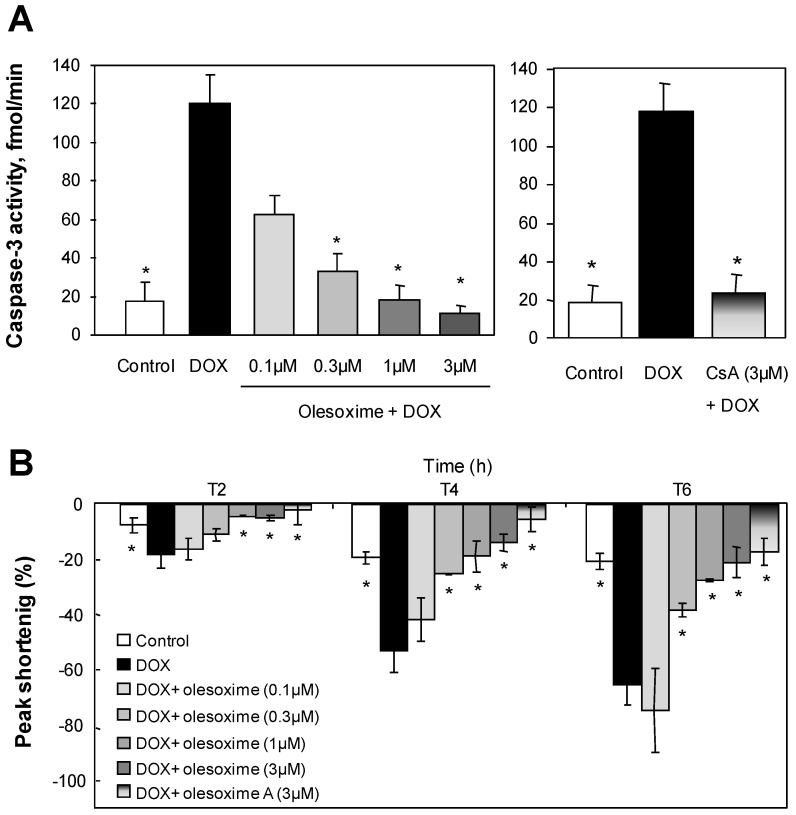
Treatment with olesoxime dose-dependently inhibits doxorubicin-induced apoptosis in adult rabbit cardiomyocytes and maintains contractility with efficacy similar to Cyclosoporin A. **(A)** Caspase-3 activity was measured in cell lysates by a fluorometric assay (Biomol Reasearch Laboratories, Plymouth Meeting, PA, USA) 8h after 1µM doxorubicin (DOX) treatment with and without olesoxime. **(B)** Time-dependent inhibition by olesoxime of doxorubicin-induced contractile dysfunction monitored by video microscopy in ventricular cardiomyocytes. Data represent mean ± SEM, n = 5-6 cells per group, * p < 0.05 *vs.* doxorubicin treated cells as determined by Student’s t-test.

While olesoxime had no effect on calcium-induced mPTP opening in isolated mitochondria, we evaluated its ability to modulate the mPTP in cells using different stress-inducers. Doxorubicin, for example, is a potent anti-neoplastic agent effective in the treatment of a wide variety of cancers. It was shown to increase mitochondrial susceptibility to both Ca^2+^ and oxidative damage [61], which is mainly responsible for its cardiotoxicity [62,63]. We evaluated olesoxime effects in a model of doxorubicin-induced apoptosis on rabbit primary cardiomyocytes [64]. At 1–3 µM, olesoxime completely protected cardiomyocyte cultures from doxorubicin toxicity as shown by a dose-dependent decrease in Annexin V labelled cells and caspase 3 activation (Figure 6 and data no shown). Normal electrical-stimulated cardiomyocyte contractility was also preserved. Olesoxime’s cytoprotective effects for cardiomyocytes were similar to those obtained by treatment with 3 µM CsA.

### 4.4. Olesoxime Rescues Hela Cells from Arichidonic Acid-Induced PTP Opening

**Figure 7:**
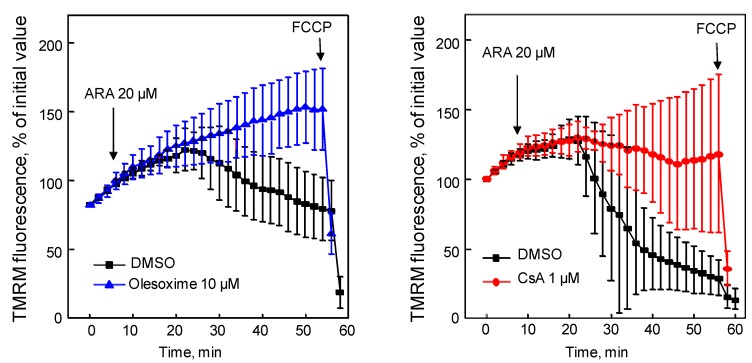
Olesoxime prevented arachidonic acid-induced mPTP opening. Hela cells were loaded with tetramethylrhodamine methyl ester (TMRM) for 5 min then treated with olesoxime or CsA (t0). Arachidonic acid (ARA) induced mitochondrial membrane depolarization measured as a loss of TMRM fluorescence in the mitochondria over time. Fluorescence intensity was measured every 2 minutes from 10 individual mitochondria. FCCP, carbonyl cyanide *p*-trifluoromethoxyphenylhydrazone. Data represent mean ± SEM.

Arachidonic acid increases ROS production and causes mPTP opening as shown by cytochrome c release, depolarization of the mitochondrial membrane, and rapid cell death when added to intact MH1C1 cells [65,66]. All these effects, which could be prevented by CsA were studied using Hela cells. As previously described in MH1C1 cells, arachidonic acid induced an extensive mitochondrial depolarization that could be prevented either by CsA or by olesoxime (Figure 7). These results demonstrate that the effects of olesoxime on stress-induced mPTP opening in rodent neurons and cardiomyocytes can also be seen in human cells subjected to oxidative stress.

### 4.5. Olesoxime Inhibits Cytochrome C Release

The anti-apoptotic properties of olesoxime were evaluated using cerebellar granule neurons (CGN) in which apoptosis is induced by incubation in a low K+ medium (5 mM KCl), as previously described [67,68]. The fraction of cytochrome C present in mitochondria was measured 15 h later by ELISA. Treatment with 3 µM olesoxime, beginning 6 h before switching to low K^+^ medium, maintained cytochrome C in mitochondrial fractions when compared to vehicle-treated neurons undergoing apoptosis [2]. 

Altogether these results suggest that olesoxime, although having little activity on isolated mitochondria, concentrates at the mitochondria and modulates mPTP opening in a variety of cell types, particularly under conditions where oxidative stress is involved. As a consequence, olesoxime efficiently prevents pro-apoptotic factor release and downstream death signaling pathways.

## 5. Early CNS Safety and Brain Penetration

**Figure 8:**
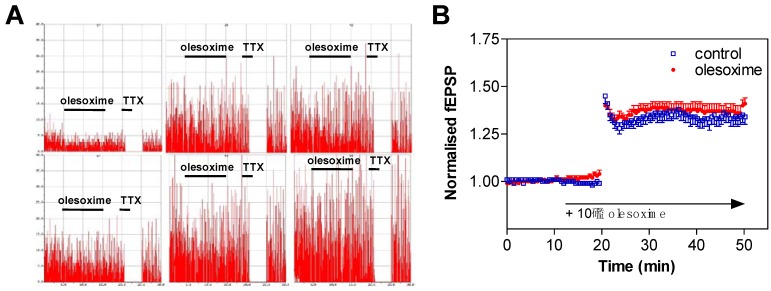
Olesoxime does not modify neuronal electrophysiological properties. (A) Spontaneous action potential was measured on rat cortical neurons exposed to 10 µM olesoxime for 10 min and then to 100 nM tetrodotoxin (TTX) for 2 min. Spike rate is plotted on a 50 Hz scale. The total session recording was 30 minutes. (B) LTP was recorded on hippocampal slices of neonatal rats in presence of 10 µM olesoxime. Data represent mean ± SEM.

Early CNS safety of olesoxime was assessed on the generation/propagation of action potentials in cultured cortical neurons. About 10 days after plating, spontaneous action potentials of cortical neurons were recorded on Multi-Electrode Arrays. The firing rate (Hz/s) was measured in control conditions (standard saline solution) for 10 minutes, and then olesoxime was perfused for an additional 10 minute period. When exposed to 10 µM olesoxime, cortical neurons did not show any modification of their firing rate. In comparison, complete firing inhibition was observed immediately after tetrodotoxin (100 nM TTX) perfusion (Figure 8A). Similarly acute exposure of rat E14 motoneurons to 10 µM olesoxime (3 min) did not modify the action potential profiles evoked by a 20 Hz stimulation (data not shown).

We next tested olesoxime effects on field excitatory action potentials (fEPSPs) evoked by stimulating the Schaeffer collaterals in the CA3 region of hippocampal slices (from 16–21 day old rats) and recorded in the CA1 region by using 3D-shaped Multi-Electrode Arrays. Most of the fEPSPs represent glutamatergic synaptic transmission [69]. They were recorded for a 10 minute period (baseline) in control conditions (O_2_-CO_2_ 95/5% buffered artificial cerebral spinal fluid; 37 °C), then olesoxime was perfused for an additional 10 minute period. No modification of the fEPSPs amplitude was observed following exposure with 10 µM olesoxime while significant fESPSPs inhibition was observed in presence of CNQX (50 µM) or TTX (100 nM). The amplitude and the stability of long term potentiation (LTP) was also not altered when hippocampal slices were exposed to 10 µM olesoxime 10 minutes before LTP induction (Figure 8B). In conclusion olesoxime does not modify the basic neuronal physiological properties of rat cortical, hippocampal and motor neurons.

Brain penetration of olesoxime was studied in mice and rats by a variety of methods. The relative level of olesoxime brain penetration compared to known brain-penetrating compounds was evaluated using the in situ rat brain perfusion technique initially developed by Q. Smith [70]. A total of six rats, 5 to 6 weeks old, were perfused with ^3^H-Olesoxime labelled in the C4 position. The permeation coefficient of the blood brain barrier (Kin) was found to be 5.9 ± 3.0 µl/g/s in a scale ranging from 0.01 to 60. When compared to reference compounds, olesoxime penetration falls between colchicine, a permeability-dependent compound, and flumazenil, a flux-dependent compound. An extraction and analytical method was also developed to detect and quantify olesoxime in both plasma and brain tissue. The amount of olesoxime in brains of mice used in various pharmacokinetic and efficacy studies allowed to compare brain levels to the AUC measured from plasma samples collected in the same study. Chronic administration performed in the nerve crush study in mice demonstrates accumulation in brain tissue over time while plasma concentrations remain constant (Table 4). Using these different approaches it can be concluded that olesoxime enters the brain and that tissue levels corresponding to efficacy can be correlated with plasma concentrations. 

**Table 4:** Olesoxime in brain and plasma after chronic administration to adult mice.

Days of treatment	Dosemg/kg/d	Plasma levelsµg/mL	Brain levelsµg/g	Brain/plasma ratio
7	0.3	0.06 ± 0.01	BLQ	-
	3	0.55 ± 0.07	0.19 ± 0.02	0.35
	30	5.35 ± 1.19	1.23 ± 0.30	0.23
42	0.3	0.06 ± 0.01	BLQ	-
	3	0.47 ± 0.01	0.24 ± 0.04	0.51
	30	5.85 ± 1.06	2.66 ± 0.84	0.45

Olesoxime was administered by subcutaneous injection in Cremophore EL/DMSO/ethanol /Phosphate Buffer Saline (5/5/10/80 v/v). Tissue and plasma samples were collected 4 h following the last administration. Values are means ± SD (n = 3 mice). BLQ, below limit of quantification.

## 6. Conclusions

Olesoxime, a molecule from a novel “cholesterol-oxime” family of cytoprotective compounds identified based on survival promoting activities on trophic factor deprived motor neurons shows neuroprotective and neuroregenerative effects in five animal models of motor nerve degeneration. Olesoxime increased motor neuron survival following facial nerve axotomy, accelerated regeneration based on EMG and histological endpoints following a sciatic nerve crush, improve CMAP parameters in chronically diabetic rats following administration of streptozotocin, delayed disease onset, prolonged survival in a transgenic model of familial ALS and increased life span in a transgenic model of SMA. Interestingly, except for studies in neonatal animals where plasma exposure levels were much higher, efficacy was correlated with doses that achieved plasma concentrations around 600 ng/mL (1.5 µM). Since efficacy is likely to be dependent on tissue concentrations and in particular, mitochondrial concentrations of the compound, the severity and rapid progression of the two neonatal animal models (axotomy-induced neurodegeneration < 1 week and SMA mice survival < 20 days post weaning) could explain why higher doses may have been necessary to rapidly attain the cellular concentrations needed to afford protection and demonstrate efficacy. Neurodegeneration and regeneration models in adult animals studied effects after at least 4 weeks of treatment with olesoxime, which may allow lower doses and plasma levels to achieve an effective tissue concentration. Given that animal models of neurodegenerative diseases and in particular the SOD1G93A model of ALS [71] may have poor predictive value for clinical efficacy, the reproducible effects of olesoxime in multiple, relevant animal models—not just SOD1G93A mice—was important for taking the decision to develop olesoxime for the treatment of motor neuron diseases.

Both binding and functional data indicate that olesoxime interacts with two outer mitochondrial membrane proteins, TSPO and VDAC, proteins implicated in control of mitochondrial permeability transition. While there are multiple models and viewpoints regarding mitochondrial permeability transition and what constitutes a mPTP complex, preventing opening of the mPTP has been shown to provide neuroprotection in different paradigms [72]. Although olesoxime does not affect calcium retention capacity of isolated mitochondria, it could be because its main effect is *via* a reduction in ROS production, which sensitizes the mPTP to opening in response to calcium. Although olesoxime inhibits mPTP opening in cells with a similar efficacy as CsA, it apparently does so by a different mechanism of action. Because it targets mitochondrial proteins, accumulates in mitochondria and prevents mitochondrial permeability transition associated with increased ROS production in rodent neurons or cardiomyocytes and human HeLa cells, we speculate that the neuroprotective effects of olesoxime might be, at least in part, due to its action on mitochondrial permeability transition. The mechanism of action of olesoxime is, however, not limited to its ability to reduce oxidative stress and mPTP opening. It also promotes neurite outgrowth and nerve repair [2], and increases neuronal microtubule dynamics (Rovini *et al*. manuscript in preparation). Therefore, olesoxime may provide additional benefits compared to mitochondrial-targeted antioxidants proposed for the treatment of ALS and other neurodegenerative disease such as creatine, triphenylphosphonium conjugated analogues of Coenzyme Q or vitamin E, or metalloporphyrins like AEOL 10150 (reviewed by [73]); although promising conceptually and showing efficacy in preclinical models, these antioxidants, where they have been tested, have unfortunately not proved efficacious in humans with neurodegenerative diseases (see [74] in this issue). 

In motor neuron diseases, early appearance of damaged mitochondria with a swollen and vacuolated appearance is a common feature within synaptic terminals [35,75,76,77], but also within skeletal muscles and the anterior horn of ALS patients [78,79,80,81,82]. Moreover, synaptic mitochondria from ALS mutant mice have reduced or defective calcium buffering capacities inducing motor nerve terminals to degenerate before spinal motoneurons begin to die [83,84]. Interestingly, an exceptionally low endogenous buffering capacity has been clearly identified as a cellular risk factor for selective motoneuron vulnerability in ALS [85]. Lastly, mitochondria may themselves be a primary disease target in familial forms of ALS linked to mutations in the SOD1 gene [86,87,88]. Thus there is reason to believe that blocking the opening of the mPTP could be a therapeutic approach to the treatment of ALS. Olesoxime acts at the level of specific mitochondrial proteins to modulate the opening of the mitochondrial pore, thus preserving essential mitochondrial functions and reducing neuronal death and degeneration. CsA, which has a related mode of action, showed protective activity in a mouse model of ALS when the blood-brain barrier had been permeabilized [89] and genetic ablation of cyclophilin D delayed disease onset and extended lifespan in the same ALS model [90] providing strong proof of principle for mPTP involvement. By contrast, olesoxime passes into the central nervous system after oral administration. In addition olesoxime displays restorative activities by promoting neurite outgrowth *in vitro* or nerve terminals *in vivo* as shown for rat motor, cortical and sensory neurons.

Olesoxime has successfully completed regulatory preclinical assessment of pharmacology, safety, toxicity and pharmacokinetics in order to conduct clinical trials. Phase 1 studies have evaluated human safety, tolerance, and pharmacokinetics of olesoxime at single and multiple doses in healthy volunteers and both ALS and SMA patients [91,92]. These clinical trials demonstrated that the product is well-tolerated and has an excellent safety profile. They also showed that once-a-day oral dosing achieves the predicted exposure level required for efficacy, based on preclinical models. Drug interaction studies with riluzole, the only registered treatment for ALS, showed no interaction of olesoxime on riluzole pharmacokinetics. Olesoxime has been granted orphan designation for the treatment of ALS and SMA both by the EMEA and FDA. Olesoxime is currently tested in a multicentre pivotal 18 month efficacy study enrolling 470 ALS patients in Europe evaluating the effects of olesoxime in combination with riluzole on survival rate as well as a number of other secondary functional endpoints. This European trial is supported by the EU MitoTarget project as part of the Seventh Framework Program of the European Community for Research, Technological Development and Demonstration Activities. Efficacy results are expected during the second half of 2011. In addition, with financial support from the Association Française contre les Myopathies, a French patient’s association supporting the discovery of therapeutics for neuromuscular diseases, a multicentre European trial in non-ambulatory SMA patients as young as 3 years old is expected to start in 2010. This study will evaluate the efficacy of olesoxime on motor function and whether electromyography parameters could serve as a potential biomarker of SMA disease progression. Hopefully these large, well-powered trials will demonstrate that olesoxime provides significant benefit to these patients.

## References

[1] Bordet T., Pruss R., Henderson C.E., Mitsumoto H., Przedborski S., Gordon P.H. (2006). Screening for ALS drugs. Amyotrophic Lateral Sclerosis.

[2] Bordet T., Buisson B., Michaud M., Drouot C., Galea P., Delaage P., Akentieva N.P., Evers A.S., Covey D.F., Ostuni M.A., Lacapere J.J., Massaad C., Schumacher M., Steidl E.M., Maux D., Delaage M., Henderson C.E., Pruss R.M. (2007). Identification and characterization of cholest-4-en-3-one, oxime (TRO19622), a novel drug candidate for amyotrophic lateral sclerosis. J. Pharmacol. Exp. Ther..

[3] Henderson C.E., Bloch-Gallego E., Camu W., Cohen J., Wilkin G. (1995). Purified embryonic motoneurons. Nerve Cell Culture: A practical approach.

[4] Greenlund L.J., Deckwerth T.L., Johnson E.M. (1995). Superoxide dismutase delays neuronal apoptosis: A role for reactive oxygen species in programmed neuronal death. Neuron.

[5] Estevez A.G., Spear N., Manuel S.M., Radi R., Henderson C.E., Barbeito L., Beckman J.S. (1998). Nitric oxide and superoxide contribute to motor neuron apoptosis induced by trophic factor deprivation. J. Neurosci..

[6] Conde C., Caceres A. (2009). Microtubule assembly, organization and dynamics in axons and dendrites. Nat. Rev. Neurosci..

[7] Valenza M., Rigamonti D., Goffredo D., Zuccato C., Fenu S., Jamot L., Strand A., Tarditi A., Woodman B., Racchi M., Mariotti C., Di Donato S., Corsini A., Bates G., Pruss R., Olson J. M., Sipione S., Tartari M., Cattaneo E. (2005). Dysfunction of the cholesterol biosynthetic pathway in Huntington's disease. J. Neurosci..

[8] Sendtner M., Kreutzberg G.W., Thoenen H. (1990). Ciliary neurotrophic factor prevents the degeneration of motor neurons after axotomy. Nature.

[9] Rossiter J.P., Riopelle R.J., Bisby M.A. (1996). Axotomy-induced apoptotic cell death of neonatal rat facial motoneurons: Time course analysis and relation to NADPH-diaphorase activity. Exp. Neurol..

[10] Vanderluit J.L., McPhail L.T., Fernandes K.J., McBride C.B., Huguenot C., Roy S., Robertson G.S., Nicholson D.W., Tetzlaff W. (2000). Caspase-3 is activated following axotomy of neonatal facial motoneurons and caspase-3 gene deletion delays axotomy-induced cell death in rodents. Eur. J. Neurosci..

[11] Tong J.X., Rich K.M. (1997). Diphenylpiperazines enhance regeneration after facial nerve injury. J. Neurocytol..

[12] Vanderluit J.L., McPhail L.T., Fernandes K.J., Kobayashi N.R., Tetzlaff W. (2003). *In vivo* application of mitochondrial pore inhibitors blocks the induction of apoptosis in axotomized neonatal facial motoneurons. Cell Death Differ..

[13] De Koning P., Brakkee J.H., Gispen W.H. (1986). Methods for producing a reproducible crush in the sciatic and tibial nerve of the rat and rapid and precise testing of return of sensory function. Beneficial effects of melanocortins. J. Neurol. Sci..

[14] Dupuis L., Loeffler J.P. (2009). Neuromuscular junction destruction during amyotrophic lateral sclerosis: Insights from transgenic models. Curr. Opin. Pharmacol..

[15] Briese M., Esmaeili B., Sattelle D.B. (2005). Is spinal muscular atrophy the result of defects in motor neuron processes?. Bioessays.

[16] Burghes A.H., Beattie C.E. (2009). Spinal muscular atrophy: Why do low levels of survival motor neuron protein make motor neurons sick?. Nat. Rev. Neurosci..

[17] Bordet T., Lesbordes J.C., Rouhani S., Castelnau-Ptakhine L., Schmalbruch H., Haase G., Kahn A. (2001). Protective effects of cardiotrophin-1 adenoviral gene transfer on neuromuscular degeneration in transgenic ALS mice. Hum. Mol. Genet..

[18] Lesbordes J.C., Cifuentes-Diaz C., Miroglio A., Joshi V., Bordet T., Kahn A., Melki J. (2003). Therapeutic benefits of cardiotrophin-1 gene transfer in a mouse model of spinal muscular atrophy. Hum. Mol. Genet..

[19] Azzouz M., Hottinger A.F., Paterna J.C., Zurn A.D., Aebischer P., Büeler H. (2000). Increased motoneuron survival and improved neuromuscular function in transgenic ALS mice after intraspinal injection of an adeno-associated virus encoding Bcl-2. Hum. Mol. Genet..

[20] Friedlander R.M., Brown R.H., Gagliardini V., Wang J., Yuan J. (1997). Inhibition of ICE slows ALS in mice. Nature.

[21] Li M., Ona V.O., Guégan C., Chen M., Jackson-Lewis V., Andrews L.J., Olszewski A.J., Stieg P.E., Lee J.P., Przedborski S., Friedlander R.M. (2000). Functional role of caspase-1 and caspase-3 in an ALS transgenic mouse model. Science.

[22] Gurney M.E., Pu H., Chiu A.Y., Dal Canto M.C., Polchow C.Y., Alexander D.D., Caliendo J., Hentati A., Kwon Y.W., Deng H.X., Chen W, Zhai P, Sufit R.L., Siddique T. (1994). Motor neuron degeneration in mice that express a human Cu, Zn superoxide dismutase mutation. Science.

[23] Pearn J. (1978). Incidence, prevalence and gene frequency studies of chronic childhood spinal muscular atrophy. J. Med. Genet..

[24] McAndrew P.E., Parsons D.W., Simard L.R., Rochette C., Ray P.N., Mendell J.R., Prior T.W., Burghes A.H. (1997). Identification of proximal spinal muscular atrophy carriers and patients by analysis of SMNT and SMNC gene copy number. Am. J. Hum. Genet..

[25] Scheffer H., Cobben J.M., Matthijs G., Wirth B. (2001). Best practice guidelines for molecular analysis in spinal muscular atrophy. Eur. J. Hum. Genet..

[26] Lefebvre S., Burglen L., Reboullet S., Clermont O., Burlet P., Viollet L., Benichou B., Cruaud C., Millasseau P., Zeviani M., Le Paslier D., Frézal J., Cohen D., Weissenbach J., Munnich A., Melki J. (1995). Identification and characterization of a spinal muscular atrophy-determining gene. Cell.

[27] Bussaglia E., Clermont O., Tizzano E., Lefebvre S., Burglen L., Cruaud C., Urtizberea J. A., Colomer J., Munnich A., Baiget M., Melki J. (1995). A frame-shift deletion in the survival motor neuron gene in Spanish spinal muscular atrophy patients. Nat. Genet..

[28] Crawford T.O., Pardo C.A. (1996). The neurobiology of childhood spinal muscular atrophy. Neurobiol. Dis..

[29] Schroth M.K. (2009). Special considerations in the respiratory management of spinal muscular atrophy. Pediatrics.

[30] Viollet L., Bertrandy S., Bueno Brunialti A.L., Lefebvre S., Burlet P., Clermont O., Cruaud C., Guenet J.L., Munnich A., Melki J. (1997). cDNA isolation, expression, and chromosomal localization of the mouse survival motor neuron gene (Smn). Genomics.

[31] DiDonato C.J., Chen X.N., Noya D., Korenberg J.R., Nadeau J.H., Simard L.R. (1997). Cloning, characterization, and copy number of the murine survival motor neuron gene: Homolog of the spinal muscular atrophy-determining gene. Genome Res..

[32] Schrank B., Götz R., Gunnersen J.M., Ure J.M., Toyka K.V., Smith A.G., Sendtner M. (1997). The inactivation of the survival motor neuron gene, a candidate gene for human spinal muscular atrophy, leads to massive cell death in early mouse embryos. Proc. Natl. Acad. Sci. USA.

[33] Hsieh-Li H.M., Chang J.G., Jong Y.J., Wu M.H., Wang N.M., Tsai C.H., Li H. (2000). A mouse model for spinal muscular atrophy. Nat. Genet..

[34] Frugier T., Tiziano F.D., Cifuentes-Diaz C., Miniou P., Roblot N., Dierich A., Le Meur M., Melki J. (2000). Nuclear targeting defect of SMN lacking the C-terminus in a mouse model of spinal muscular atrophy. Hum. Mol. Genet..

[35] Cifuentes-Diaz C., Nicole S., Velasco M.E., Borra-Cebrian C., Panozzo C., Frugier T., Millet G., Roblot N., Joshi V., Melki J. (2002). Neurofilament accumulation at the motor endplate and lack of axonal sprouting in a spinal muscular atrophy mouse model. Hum. Mol. Genet..

[36] Authier N., Gillet J.P., Fialip J., Eschalier A., Coudore F. (2000). Description of a short-term Taxol-induced nociceptive neuropathy in rats. Brain Res..

[37] Authier N., Gillet J.P., Fialip J., Eschalier A., Coudore F. (2003). A new animal model of vincristine-induced nociceptive peripheral neuropathy. Neurotoxicology.

[38] Thant M., Hawley R.J., Smith M.T., Cohen M.H., Minna J.D., Bunn P.A., Ihde D.C., West W., Matthews M.J. (1982). Possible enhancement of vincristine neuropathy by VP-16. Cancer.

[39] Polomano R.C., Mannes A.J., Clark U.S., Bennett G.J. (2001). A painful peripheral neuropathy in the rat produced by the chemotherapeutic drug, paclitaxel. Pain.

[40] Flatters S.J., Bennett G.J. (2004). Ethosuximide reverses paclitaxel- and vincristine-induced painful peripheral neuropathy. Pain.

[41] Flatters S.J., Bennett G.J. (2006). Studies of peripheral sensory nerves in paclitaxel-induced painful peripheral neuropathy: Evidence for mitochondrial dysfunction. Pain.

[42] Flatters S.J., Xiao W.H., Bennett G.J. (2006). Acetyl-L-carnitine prevents and reduces paclitaxel-induced painful peripheral neuropathy. Neurosci Lett.

[43] Siau C., Bennett G.J. (2006). Dysregulation of cellular calcium homeostasis in chemotherapy-evoked painful peripheral neuropathy. Anesth. Analg..

[44] Xiao W., Bennett G.J. (2008). Chemotherapy-evoked neuropathic pain: Abnormal spontaneous discharge in A-fiber and C-fiber primary afferent neurons and its suppression by acetyl-L-carnitineitine. Pain.

[45] Xiao W.H., Zheng F.Y., Bennett G.J., Bordet T., Pruss R.M. (2009). Olesoxime (cholest-4-en-3-one, oxime): Analgesic and neuroprotective effects in a rat model of painful peripheral neuropathy produced by the chemotherapeutic agent, paclitaxel. Pain.

[46] Ahlgren S.C., Levine J.D. (1993). Mechanical hyperalgesia in streptozotocin-diabetic rats. Neuroscience.

[47] Courteix C., Eschalier A., Lavarenne J. (1993). Streptozocin-induced diabetic rats: Behavioural evidence for a model of chronic pain. Pain.

[48] Calcutt N.A., Li L., Yaksh T.L., Malmberg A.B. (1995). Different effects of two aldose reductase inhibitors on nociception and prostaglandin E. Eur. J. Pharmacol..

[49] Calcutt N.A., Jorge M.C., Yaksh T.L., Chaplan S.R. (1996). Tactile allodynia and formalin hyperalgesia in streptozotocin-diabetic rats: Effects of insulin, aldose reductase inhibition and lidocaine. Pain.

[50] Malcangio M., Tomlinson D.R. (1998). A pharmacologic analysis of mechanical hyperalgesia in streptozotocin/diabetic rats. Pain.

[51] Field M.J., McCleary S., Hughes J., Singh L. (1999). Gabapentin and pregabalin, but not morphine and amitriptyline, block both static and dynamic components of mechanical allodynia induced by streptozocin in the rat. Pain.

[52] Cameron N.E., Cotter M.A., Low P.A. (1991). Nerve blood flow in early experimental diabetes in rats: Relation to conduction deficits. Am. J. Physiol..

[53] Jakobsen J. (1976). Axonal dwindling in early experimental diabetes. II. A study of isolated nerve fibres. Diabetologia.

[54] Chen X., Levine J.D. (2001). Hyper-responsivity in a subset of C-fiber nociceptors in a model of painful diabetic neuropathy in the rat. Neuroscience.

[55] Chen X., Levine J.D. (2003). Altered temporal pattern of mechanically evoked C-fiber activity in a model of diabetic neuropathy in the rat. Neuroscience.

[56] Orstavik K., Namer B., Schmidt R., Schmelz M., Hilliges M., Weidner C., Carr R.W., Handwerker H., Jorum E., Torebjork H.E. (2006). Abnormal function of C-fibers in patients with diabetic neuropathy. J. Neurosci..

[57] Bordet T., Buisson B., Michaud M., Abitbol J.L., Marchand F., Grist J., Andriambeloson E., Malcangio M., Pruss R.M. (2008). Specific antinociceptive activity of cholest-4-en-3-one, oxime (TRO19622) in experimental models of painful diabetic and chemotherapy-induced neuropathy. J. Pharmacol. Exp. Ther..

[58] Leung A.W., Halestrap A.P. (2008). Recent progress in elucidating the molecular mechanism of the mitochondrial permeability transition pore. Biochim. Biophys. Acta.

[59] Rasola A., Bernardi P. (2007). The mitochondrial permeability transition pore and its involvement in cell death and in disease pathogenesis. Apoptosis.

[60] Waldmeier P.C., Zimmermann K., Qian T., Tintelnot-Blomley M., Lemasters J.J. (2003). Cyclophilin D as a drug target. Curr. Med. Chem..

[61] Cardoso S., Santos R.X., Carvalho C., Correia S., Pereira G.C., Pereira S.S., Oliveira P.J., Santos M.S., Proenca T., Moreira P.I. (2008). Doxorubicin increases the susceptibility of brain mitochondria to Ca(2+)-induced permeability transition and oxidative damage. Free Radic. Biol. Med..

[62] Yen H.C., Oberley T.D., Vichitbandha S., Ho Y.S., St Clair D.K. (1996). The protective role of manganese superoxide dismutase against adriamycin-induced acute cardiac toxicity in transgenic mice. J. Clin. Invest.

[63] Berthiaume J.M., Wallace K.B. (2007). Adriamycin-induced oxidative mitochondrial cardiotoxicity. Cell Biol. Toxicol..

[64] d'Anglemont de Tassigny A., Berdeaux A., Souktani R., Henry P., Ghaleh B. (2008). The volume-sensitive chloride channel inhibitors prevent both contractile dysfunction and apoptosis induced by doxorubicin through PI3kinase, Akt and Erk 1/2. Eur. J. Heart Fail.

[65] Scorrano L., Penzo D., Petronilli V., Pagano F., Bernardi P. (2001). Arachidonic acid causes cell death through the mitochondrial permeability transition. Implications for tumor necrosis factor-alpha aopototic signaling. J. Biol. Chem..

[66] Petronilli V., Penzo D., Scorrano L., Bernardi P., Di Lisa F. (2001). The mitochondrial permeability transition, release of cytochrome c and cell death. Correlation with the duration of pore openings *in situ*. J. Biol. Chem..

[67] Schousboe A., Meier E., Hertz L., Shaher A., DeVellis J., Vernadakis A., Haber B., Shahar A.D., De Vellis J., Vernadakis A., Haber B. (1989). Preparation of cultures of mouse (rat) cerebellar granule cells. Dissection and Tissue Culture Manual of the Nervous System.

[68] D'Mello S.R., Galli C., Ciotti T., Calissano P. (1993). Induction of apoptosis in cerebellar granule neurons by low potassium: Inhibition of death by insulin-like growth factor I and cAMP. Proc. Natl. Acad. Sci. USA.

[69] Steidl E.M., Neveu E., Bertrand D., Buisson B. (2006). The adult rat hippocampal slice revisited with multi-electrode arrays. Brain Res..

[70] Takasato Y., Rapoport S.I., Smith Q.R. (1984). An *in situ* brain perfusion technique to study cerebrovascular transport in the rat. Am. J. Physiol..

[71] Scott S., Kranz J.E., Cole J., Lincecum J.M., Thompson K., Kelly N., Bostrom A., Theodoss J., Al-Nakhala B.M., Vieira F.G., Ramasubbu J., Heywood J.A. (2008). Design, power, and interpretation of studies in the standard murine model of ALS. Amyotroph Lateral Scler.

[72] Mattson M.P., Kroemer G. (2003). Mitochondria in cell death: Novel targets for neuroprotection and cardioprotection. Trends Mol. Med..

[73] Szeto H.H. (2006). Mitochondria-targeted peptide antioxidants: Novel neuroprotective agents. Aaps. J..

[74] Swerdlow R.H. (2009). Mitochondrial medicine and the neurodegenerative mitochondriopathies. Pharmaceuticals.

[75] Wong P.C., Pardo C.A., Borchelt D.R., Lee M.K., Copeland N.G., Jenkins N.A., Sisodia S.S., Cleveland D.W., Price D.L. (1995). An adverse property of a familial ALS-linked SOD1 mutation causes motor neuron disease characterized by vacuolar degeneration of mitochondria. Neuron.

[76] Kong J., Xu Z. (1998). Massive mitochondrial degeneration in motor neurons triggers the onset of amyotrophic lateral sclerosis in mice expressing a mutant SOD1. J. Neurosci..

[77] Acsadi G., Lee I., Li X., Khaidakov M., Pecinova A., Parker G.C., Huttemann M. (2009). Mitochondrial dysfunction in a neural cell model of spinal muscular atrophy. J. Neurosci. Res..

[78] Afifi A.K., Aleu F.P., Goodgold J., MacKay B. (1966). Ultrastructure of atrophic muscle in amyotrophic lateral sclerosis. Neurology.

[79] Atsumi T. (1981). The ultrastructure of intramuscular nerves in amyotrophic lateral sclerosis. Acta Neuropathol (Berl).

[80] Hirano A., Nakano I., Kurland L.T., Mulder D.W., Holley P.W., Saccomanno G. (1984). Fine structural study of neurofibrillary changes in a family with amyotrophic lateral sclerosis. J. Neuropathol. Exp. Neurol..

[81] Sasaki S., Iwata M. (1996). Ultrastructural study of synapses in the anterior horn neurons of patients with amyotrophic lateral sclerosis. Neurosci. Lett.

[82] Echaniz-Laguna A., Zoll J., Ponsot E., N'Guessan B., Tranchant C., Loeffler J.P., Lampert E. (2006). Muscular mitochondrial function in amyotrophic lateral sclerosis is progressively altered as the disease develops: A temporal study in man. Exp. Neurol..

[83] Vila L., Barrett E.F., Barrett J.N. (2003). Stimulation-induced mitochondrial [Ca2+] elevations in mouse motor terminals: Comparison of wild-type with SOD1-G93A. J. Physiol..

[84] David G., Barrett E.F. Quantal release from motor nerve terminals of mice expressing the G93A mutation of human superoxide dismutase 1 (SOD1G93A). Neuroscience Meeting.

[85] Lewinski F.V., Keller B.U. (2005). Ca(2+), mitochondria and selective motoneuron vulnerability: Implications for ALS. Trends Neurosci..

[86] Mattiazzi M., D'Aurelio M., Gajewski C.D., Martushova K., Kiaei M., Beal M.F., Manfredi G. (2002). Mutated human SOD1 causes dysfunction of oxidative phosphorylation in mitochondria of transgenic mice. J. Biol. Chem..

[87] Liu J., Lillo C., Jonsson P.A., Vande Velde C., Ward C.M., Miller T.M., Subramaniam J.R., Rothstein J.D., Marklund S., Andersen P.M., Brannstrom T., Gredal O., Wong P.C., Williams D.S., Cleveland D.W. (2004). Toxicity of familial ALS-linked SOD1 mutants from selective recruitment to spinal mitochondria. Neuron.

[88] Pasinelli P., Belford M.E., Lennon N., Bacskai B.J., Hyman B.T., Trotti D., Brown R.H. (2004). Amyotrophic lateral sclerosis-associated SOD1 mutant proteins bind and aggregate with Bcl-2 in spinal cord mitochondria. Neuron.

[89] Kirkinezos I.G., Hernandez D., Bradley W.G., Moraes C.T. (2004). An ALS mouse model with a permeable blood-brain barrier benefits from systemic cyclosporine A treatment. J. Neurochem..

[90] Martin L.J., Gertz B., Pan Y., Price A.C., Molkentin J.D., Chang Q. (2009). The mitochondrial permeability transition pore in motor neurons: Involvement in the pathobiology of ALS mice. Exp. Neurol..

[91] Abitbol J.L., Cuvier V., Bordet T., Drouot C., Berna P., Pruss R.M. Safety and pharmacokinetics of repeated doses of TRO19622, a drug candidate for the treatment of amyotrophic lateral sclerosis and spinal muscular atrophy. ALS/MND International Symposium.

[92] Estournet B., Chabrol B., Cuisset J.M., Cances C., Strub-Wourgaft N., Cuvier V., Bassissi F., Bordet T., Pruss R., Abitbol J.L. Safety and pharmacokinetics (PK) of TRO19622 in Spinal Muscular Atrophy (SMA) children and adults. 61st Annual Meeting American Academy of Neurology.

